# SARS-CoV-2 Accessory Proteins in Viral Pathogenesis: Knowns and Unknowns

**DOI:** 10.3389/fimmu.2021.708264

**Published:** 2021-07-07

**Authors:** Natalia Redondo, Sara Zaldívar-López, Juan J. Garrido, Maria Montoya

**Affiliations:** ^1^ Molecular Biomedicine Department, Centro de Investigaciones Biológicas Margarita Salas (CIB-CSIC), Madrid, Spain; ^2^ Animal Breeding and Genomics Group, Department of Genetics, Faculty of Veterinary Medicine, University of Córdoba, Córdoba, Spain; ^3^ Immunogenomics and Molecular Pathogenesis GA14 Group, Maimónides Biomedical Research Institute of Córdoba (IMIBIC), Córdoba, Spain

**Keywords:** SARS-CoV-2, accessory proteins, COVID-19, immune response, coronavirus

## Abstract

There are still many unanswered questions concerning viral SARS-CoV-2 pathogenesis in COVID-19. Accessory proteins in SARS-CoV-2 consist of eleven viral proteins whose roles during infection are still not completely understood. Here, a review on the current knowledge of SARS-CoV-2 accessory proteins is summarized updating new research that could be critical in understanding SARS-CoV-2 interaction with the host. Some accessory proteins such as ORF3b, ORF6, ORF7a and ORF8 have been shown to be important IFN-I antagonists inducing an impairment in the host immune response. In addition, ORF3a is involved in apoptosis whereas others like ORF9b and ORF9c interact with cellular organelles leading to suppression of the antiviral response in infected cells. However, possible roles of ORF7b and ORF10 are still awaiting to be described. Also, ORF3d has been reassigned. Relevant information on the knowns and the unknowns in these proteins is analyzed, which could be crucial for further understanding of SARS-CoV-2 pathogenesis and to design strategies counteracting their actions evading immune responses in COVID-19.

## Highlights

- SARS-CoV-2 accessory proteins play important roles in pathogenesis
- SARS-CoV-2 accessory proteins ORF3b, ORF6, ORF7a and ORF8 are potent interferon antagonists
- SARS-CoV-2 accessory proteins impaired host immune response through different mechanisms
- Accessory proteins can be potential targets for the development of new treatments against Covid-19

## Introduction

The coronavirus disease 2019 (COVID-19) is a potentially fatal respiratory disease caused by a new severe acute respiratory syndrome coronavirus 2 (SARS-CoV-2), which was first identified in December 2019 in Wuhan (Hubei Province, China). Since then, it has rapidly spread worldwide, causing more than 150 million reported cases and over 3,1 million deaths globally since the start of the pandemic (WHO, May 4 situation report). COVID-19 has become the leading cause of morbidity and mortality in many countries and exemplifies the devastating impact of an emerging zoonotic pathogen on global public health and socio-economic development. The clinical course of COVID-19 exhibits a broad spectrum of severity and progression patterns. While in a significant number of people SARS-CoV-2 infection leads to mild upper respiratory disease or even asymptomatic sub-clinical infection, others develop symptoms and complications of severe pneumonia that can be fatal (1). Acute respiratory distress syndrome (ARDS), pulmonary edema, acute kidney injury, severe sepsis with shock, and even multiple organ failure are associated with the highest rates of mortality (2). Since March 2020 many efforts have been done to ascertain and elucidate COVID-19 pathogenesis, however the complete clinical picture following SARS-CoV-2 infection is not yet fully understood although it is known that the underlying cause of severe disease is a cytokine dysregulation and hyperinflammation status triggered by an impaired in interferon responses (3).

Taxonomically, SARS CoV-2 belongs to the *Coronaviridae* family of order *Nidovirales*, which is divided into four genera on the basis of genetic and serologic properties, Alphacoronavirus, Betacoronavirus, Gammacoronavirus, and Deltacoronavirus (4). *Betacoronavirus* genus includes SARS-CoV-2 and also other pathogenic coronaviruses transmitted through zoonotic transmission such as severe acute respiratory syndrome coronavirus (SARS-CoV), and Middle East respiratory syndrome coronavirus (MERS-CoV). Whereas MERS-CoV is a member of the Merbecovirus subgenus, phylogenetic analyses indicated that SARS-CoV-2 clusters with SARS-CoV in the Sarbecovirus subgenus. SARS-CoV-2 genome shares about 82% genome identity with SARS-CoV and ≈50% genome identity with MERS-CoVsupporting the idea of common mechanisms. Nevertheless, SARS-CoV-2 has resulted in the most devastating pandemic of the twenty-first century, comparing with the recent coronavirus outbreaks caused by SARS-CoV (2002-2003) or MERS-CoV (2012) (5).

**Figure 1:**
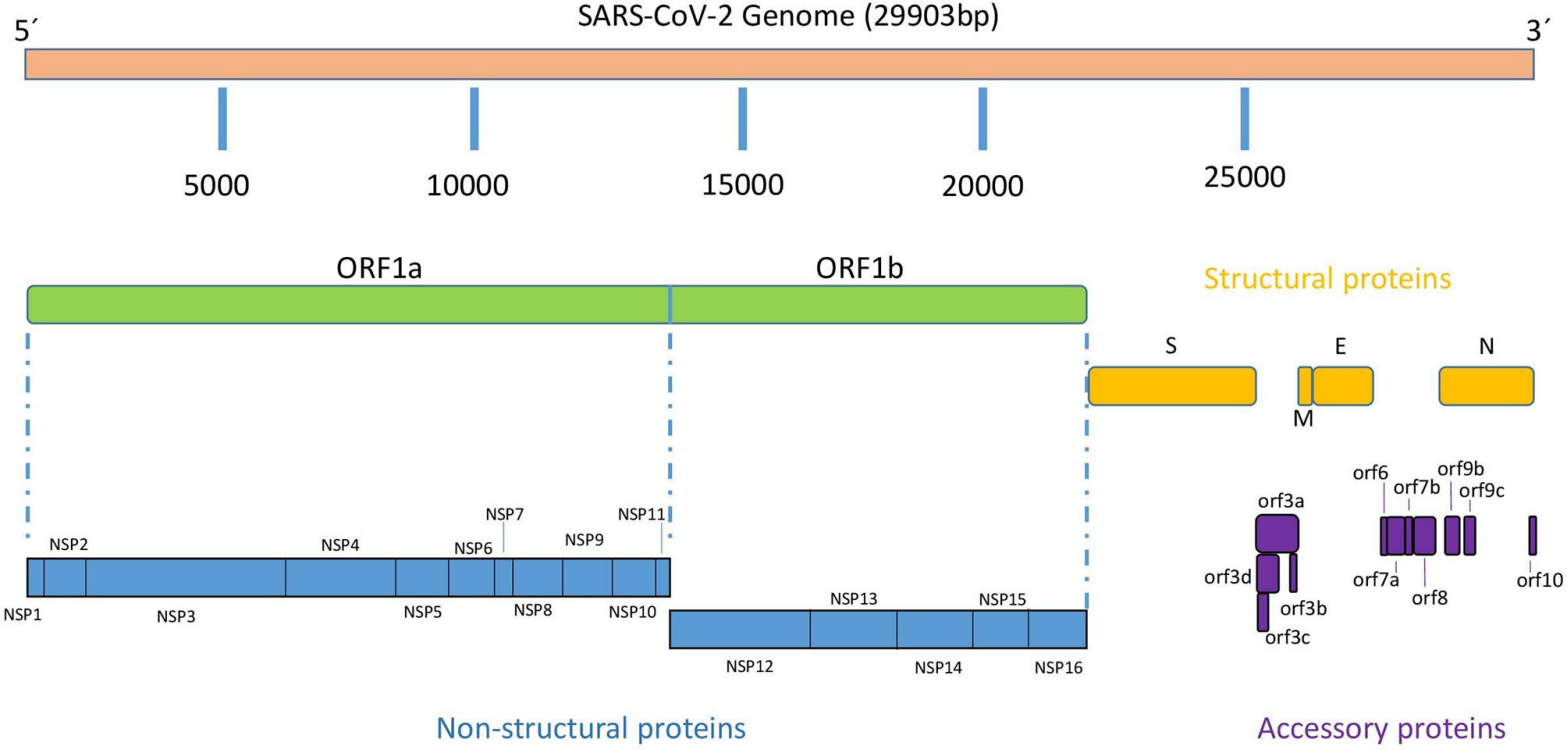
SARS-CoV-2 genomic organization.

Similar to the rest of *Coronavirus*es, the SARS-CoV-2 genome consists of a single-stranded positive-sense RNA molecule of approximately 29,900 nucleotides (NCBI Reference Sequence: NC_045512.2) arranged into 14 open reading frames (ORFs) encoding 31 proteins as shown schematically in **Figure 1** (6). Following a typical 5’-3’ order of appearance, SARS-CoV-2 proteins comprise two large polyproteins: ORF1a and ORF1ab that proteolytically cleaves by a virus-encoded protease into individual replicase complex nonstructural proteins to form 16 non-structural proteins (nsp1-16) involved in genome replication and early transcription regulation (7); four structural proteins: spike (S), envelope (E), membrane (M), and nucleocapsid (N), which are common to all coronaviruses and are considered to be major therapeutic targets for antiviral drug development (8); and eleven accessory proteins: ORF3a, ORF3b, ORF3c, ORF3d, ORF6, ORF7a, ORF7b, ORF8, ORF9b, ORF9c and ORF10 (9). SARS-CoV-2 life cycle replication begins in virus-induced double- membrane vesicles derived from the endoplasmic reticulum (ER), which ultimately integrate to form elaborate webs of convoluted membranes. Here, the incoming positive-strand genome then serves as a template for full-length negative-strand RNA and subgenomic (sg) RNA. sgRNA translation results in both structural proteins and accessory proteins (10).

The coronavirus genome is unique among *Nidoviruses* because it encodes a variable number of accessory proteins whose function appears not essential for virus replication but seem to play a relevant role in pathogenesis. In the case of SARS-CoV-2, accessory proteins have been less characterized than other proteins contained in the viral genome, and much of their understanding is based on extensive functional studies reported on SARS-CoV and other related viruses such as MERS-CoV (11). Although these proteins are not essential for virus replication, it is known that some have been shown to play an important role in virus-host interactions (12). Evolutionary analysis indicated that some of these accessory proteins were dominating the early evolutionary trends of SARS-CoV-2 (13). Furthermore, mutations in accessory proteins such as ORF3a, ORF6, ORF7a, ORF8 or ORF10 have been observed in currently circulating “variants of concern” thus potentially contributing to increasing pathogenesis and transmissibility in these SARS-CoV-2 strains (https://covariants.org/variants).

Here, the current knowledge of SARS-CoV-2 accessory proteins is reviewed with special emphasis on what is currently known and unknown about their involvement in the pathological inflammatory responses in COVID-19.

## Accessory Proteins of SARS-CoV-2

### ORF3a

ORF3a is the largest among all accessory proteins encoded by SARS-CoV-2, with 275 aa residues in length, sharing 72,7% protein identity with the SARS-CoV ORF3a (**Figure 1**) (14, 15). Structurally, this protein is a viroporin, an integral membrane protein able to function as an ion channel that may promote virus release (16) and it is required for maximal replication and virulence (17). ORF3a protein possess a cysteine-rich domain, a tyrosine-based sorting motif (YXXΦ) and a diacidic EXD motif, all of them involved in the cellular distribution of the cell, internalization of proteins into different subcellular compartments or in pathogenesis and immune evasion (18, 19). Cysteine-rich motif and YXXΦ motif are conserved in SARS-CoV-2 but not the EXD motif. Functionally, it has been shown that ORF3a interacts with the host immune system by activating pro-IL-1β gene expression and IL-1β secretion, thereby activating ultimately NF-kB signaling and NLRP3 inflammasomes and promoting the generation of cytokine storms (20, 21). In addition, SARS-CoV ORF3a has been described to induce necrotic cell death and lysosomal damage (22).

The ability of SARS-CoV-2 ORF3a to cause apoptosis in cells has also been described by Ren and co-workers (23). ORF3a induced apoptosis *via* caspase-3 activation when expressed in Vero, HEK293T, and HepG2 cells. Also, they established that ORF3a activates the extrinsic apoptotic pathway in which caspase-8 cleaves Bid to tBid, releasing cytochrome c from the mitochondria and leading to apoptosome formation and caspase-9 activation. Ren and colleagues construct two defective mutants in membrane binding to investigate the relation between ORF3a proapoptotic activity and its plasmatic membrane attachment. They found that these two mutants were unable to induce apoptosis thus concluding that binding of ORF3a protein to the membrane is necessary for its pro-apoptotic activity (15). Finally, interaction of SARS-CoV-2 ORF3a with the lysosomal pathway has been described, impairing autophagocytic activity and presumably inducing lysosomal evasion (24). Their mechanism would imply sequestration of homotypic fusion and protein sorting (HOPS) component VPS39 which subsequently avoids the interaction between HOPS complex and autophagosomal SNARE protein STX17. All in all, ORF3a’s main interactions with the cell machinery have been described in inflammation, with IL1β secretion and NLRP3 activation, and in apoptosis.

### ORF3b

ORF3b protein is 22 aa in length (69 bp, including stop codon) (**Figure 1**), significantly shorter than its homologous in SARS-CoV, which is approximately 153 aa long on average. Despite its small size, Konno and co-workers have shown that SARS-CoV-2 ORF3b protein is a potent interferon (IFN) antagonist, suppressing the induction of type I interferon more efficiently than its SARS-CoV ortholog (25). Interestingly, phylogenetic studies have shown ORF3b similar characteristics in SARS-CoV-2-related viruses found in other species such as pangolins and bats. The efficacy of IFN antagonism by ORF3b appears to be related with the length of the C terminus and the subcellular location of the protein and both with the ability of SARS-CoV-2 ORF3b to hamper the nuclear translocation of IRF3, a transcription factor key regulator of IFNB1 expression. Thus, SARS-CoV-2 ORF3b proteins showing significant activity against IFN-I are mainly localized in the cytosol, whereas their less active SARS-CoV homologues are found in both the cytosol and the nucleus.

### ORF3c

ORF3c (originally known as iORF1) is a 41-codon long protein recently described as an accessory protein (**Figure 1**) (26, 27). ORF3c is encoded by a frame overlapping *ORF3a* (close to the 5’ end) and is conserved across *Sarbecoviruses*. It has a predicted highly conserved transmembrane domain, which suggests interactions within the lipid bilayer such as membrane-disrupting or membrane-associated signaling activities. Ribosomal profiling by Ribo-seq confirmed that ORF3c is translated during infection, but future studies are necessary to investigate the unknown role of this protein during SARS-CoV-2 infection (28).

### ORF3d

Overlapping genes are a form of genomic evolution and innovation, where a set of nucleotides can code for different proteins. *ORF3d* has been recently described in SARS-CoV-2 as a new overlapping gene encoding a 57 aa long protein (29). *ORF3d* maps at the 5’ end of the *ORF3a* sequence overlapping the 3’ half of *ORF3c*. Since it was first described as *ORF3b* by Chan et al. (30), literature must be depicted carefully since some reports may have conflated these two ORFs, interchanging the names (31). Nowadays, we know that *ORF3b* and *ORF3d* are unrelated and located in different genomic positions (**Figure 1**). Phylogenetic analysis showed that *ORF3d* is restricted to a subset of *Betacoronaviruses*, suggesting that this ORF can be used to study taxonomic differences between coronaviruses. Gordon and colleagues demonstrated ORF3d protein interaction with mitochondrial protein STOML2, although it was erroneously considered ORF3b in their paper (32). No other functional features have been described to our knowledge, leaving a substantial gap of knowledge for ORF3d function on SARS-CoV-2 infection and pathogenesis. Nevertheless, together with N and ORF8 proteins, ORF3d elicits the strongest antibody responses measured in sera from COVID-19 patients (33).

### ORF6

The *ORF6* gene is common to all *Sarbecoviruses*, including SARS-CoV and SARS-CoV-2, and no orthologues have been found in other *Betacoronaviruses*, such as MERS-CoV. The protein encoded by SARS-CoV-2 *ORF6* gene is 61 aa residues and has been localized to the endoplasmic reticulum and membrane of vesicles such as autophagosomes and lysosomes (34). SARS-CoV-2 ORF6 protein, is a potent IFN antagonist, a role that was previously described by Kopecky-Bromberg and coworkers in SARS-CoV (35).

The mechanism leading to suppression of the IFN responses by SARS-CoV-2 ORF6 was elucidated by Miorin et al. According to these authors, ORF6 is able to block transcription factor STAT transportation from the cytoplasm to the nucleus, blocking IFN activation (36). As previously shown by other viruses´ proteins, such as matrix (M) protein in vesicular stomatitis virus (VSV), SARS-CoV-2 ORF6 mechanism seems to be based on binding to Rae1 and Nup98, thus forming a complex that inhibits cytoplasm-nucleus trafficking. This mechanism was similarly described in SARS-CoV where ORF6 protein was able to inhibit STAT1 nuclear transportation (35, 37, 38). Presumably, the mechanism whereby ORF6 binds Rae1-Nup98 would be similar to the M protein binding in VSV but it remains to be explored. In this context, Met58 in ORF6 would play a critical role since structural differences are evident between both proteins (36, 39). In line with this nuclear-cytoplasm trafficking inhibition, Kato and collaborators evidenced accumulation of heterogeneous ribonucleoprotein A (hnRNPA1) in the nucleus when expressing ORF6 protein when compared to their control counterparts (40).

### ORF7a

SARS-CoV-2 ORF7b is a type-I transmembrane protein of 121 aa residues (**Figure 1**) with an N-terminal signal peptide (15 residues), an ectodomain, a transmembrane region, and a cytoplasmic di-lysine motif (KRKTE) for ER localization. The structure shows a compact seven-stranded β-sandwich fold similar to the immunoglobulin superfamily (41). This protein shares 85.2% identity and 95.9% sequence similarity with ORF7a protein from SARS-CoV (7). ORF7a is another SARS-CoV-2 protein with the ability to antagonize the IFN-I response (42). Thus, Cao and colleagues showed that SARS-CoV-2 hijacks the host ubiquitin system to enhance ORF7a’s ability to antagonize IFN-I responses (43). Protein ubiquitination is a post-translational modification that can regulate many aspects of eukaryotic biology, including viral infections. Some other SARS-CoV-2 proteins with IFN-I antagonism, such as nsp13 and ORF3a also appear to be ubiquitinated. ORF7a is polyubiquitinated at position Lys 119 (K119) and this might lead to inhibition of IFN-I response *via* blocking of STAT2 (38). A recent study has shown that SARS-CoV-2 ORF7a ectodomain binds to CD14+ monocytes in human peripheral blood with high efficiency, leading to decreased antigen-presenting ability and inducing a dramatic expression of proinflammatory cytokines by human immune cells (44). Since the lung is the one of main site of SARS-CoV-2 replication, interaction between SARS-CoV-2 ORF7a and monocytes suggests that this protein can play a pivotal function in the recruitment of monocytes to the lung during COVID-19.

### ORF7b

SARS-CoV-2 ORF7b protein is 43 aa residues long (**Figure 1**), one aa less than that in SARS-CoV. Both proteins share 85.4% identity and 97.2% sequence similarity (7). Some reports have shown that the homologous ORF7b protein in SARS-CoV is a transmembrane protein localized in the Golgi apparatus not essential for virus replication (11, 45). There is little data on the ORF7b protein of SARS-CoV-2 far beyond the evidence that SARS-CoV-2 strains collected from clinical samples have shown deletions in ORF7a and ORF8 leading to the formation of an ORF7b-ORF8 fusion protein with a truncated ORF7b and the C-terminus of ORF8 (46). Because ORF7b functional analysis remains to be performed, some authors have shown that this protein assembles into stable multimers through a leucine zipper. They have hypothesized that ORF7b could potentially interfere with some cellular processes that underlie some common symptoms of SARS-CoV-2 infection involving leucine zipper formation and epithelial cell-cell adhesion, such as heart rate dysregulation (47).

### ORF8

*ORF8* gene in SARS-CoV-2 is 366 nucleotides long poorly conserved among coronavirus, showing structural plasticity and high diversity that has been suggested to play an important role in pathogenesis (48). *ORF8* gene is unique to SARS-CoV-2 and appears to have been generated by differentiation from a fragmented *ORF8a/b* sequence present in other coronaviruses such as SARS-CoV (49, 50). The protein encoded by SARS-CoV-2 *ORF8* gene is a 121-amino acid protein (**Figure 1**) consisting of an N-terminal signal sequence for endoplasmic reticulum (ER) import, followed by a predicted Ig-like fold to interact with a variety of host proteins, including many factors involved in ER-associated degradation and vesicle trafficking (32, 51). ORF8 is a secreted protein, rather than retained in the ER, and its extracellular form has been detected in the supernatant of cell cultures and in the sera of COVID-19 patients. In addition, ORF8 was found to be highly immunogenic in COVID-19 patients and therefore, can be used for accurate diagnosis of COVID-19. Indeed, the immunogenicity of ORF8 is supported by the fact that, together with N protein and ORF3d, ORF8 elicits the strongest and most specific antibody response among the SARS-CoV-2 antigens, both in the mid and late phases and in early infection (32, 52).

The exact function of ORF8 is still elusive. Nevertheless, it has been shown that deletions in ORF8 result in milder disease, decreased hypoxia and decreased release of inflammatory cytokines in SARS-CoV-2 infections (53). Several important functions have been attributed to SARS-CoV-2 ORF8, including apoptosis (54) and antagonizing the IFN signaling pathway (55). Also, complete absence of ORF8 reduced replicative capacity of SARS-CoV (56). In SARS-CoV-2, functional studies have begun to uncover a role for ORF8 in evading host innate immunity processes by impairing the antigen presentation and type I IFN-mediated antiviral response. On one side, and consistent with the idea of its accumulation in the ER, SARS-CoV-2 ORF8, can directly interact with class I major histocompatibility complex molecules (MHC-I) and significantly down-regulates their surface expression on various cell types (57). On the other side, SARS-CoV-2 ORF8 is a type I IFN antagonist, which exerts its function by targeting the PRRs-mediated pathway of IFN-β promoter and the downstream signal transduction that induces the interaction between IFN-β and IFNAR of INF stimulated genes (58). Also, ORF8 could activate IL-17 signaling pathway and promote proinflammatory factors expression by interacting with host IL17RA, thus contributing to SARS-CoV-2 cytokine storm during infection (59).

### ORF9b

ORF9b is an alternative ORF located within the nucleocapsid (N) gene (**Figure 1**), which codes for a 97 aa long protein localized in the mitochondrial membrane. In SARS-CoV, ORF9b suppresses innate immunity by targeting MAVS signalosome, with subsequent loss of TRAF3 and TRAF6, therefore limiting host interferon response. ORF9 overexpression induced autophagy in host cells mediated by ATG5 (60). A study of protein interactions showed that Tom70 (encoded by *TOMM70*), a mitochondrial import receptor, forms a complex with ORF9b both in SARS-CoV and SARS-CoV-2 (32). This complex may modulate the host immune response by compromising type I IFN synthesis (61). When its RNA enters host mitochondria, SARS-CoV-2 manipulates this organelle in various ways. Examples of this manipulation are ACE2 overexpression and binding to S protein to gain entry into the cell, and ORF9b-mediated activation of inflammasome to evade immune responses facilitating viral replication (62). In some cases, significant antibody responses to ORF9b have been found in COVID-19 convalescent patients (63).

### ORF9c

ORF9c shares 94% sequence identity with bat SARS-coronavirus ORF14, and 74% with SARS-CoV ORF14. In SARS-CoV-2, a transmembrane domain has been described in this protein (64). Gordon, Jang et al., 2020 found that ORF9c interacts with Sigma receptors that are implicated in lipid remodeling and ER stress response. Also, they found evidence of protein interaction between ORF9c protein and NF-kB related molecules such as Nod-like receptor NLRX1, proteinase-activated receptor 2 (F2RL1), and Nedd4 Family Interacting Protein 2 (NDFIP2) (32). Experimental studies have shown that ORF9c SARS-CoV-2 suppresses antiviral response. Particularly, ORF9c expression impaired interferon signaling, antigen processing and presentation, complement signaling, and induced IL-6 signaling (64).

### ORF10

A hypothetical 38aa long protein has been described as ORF10. Perhaps this one is the least attractive of the accessory proteins, since it has been shown that it is not essential in human SARS-CoV-2 infection (65). Its sequence is not similar to other coronavirus, and specific biological functions have not been described yet. Deletion of ORF10 does not change replication ability of SARS-CoV-2, and transmission is similar. However, mutations leading to lower stability of protein have been described (66).

## Conclusions

**Figure 2:**
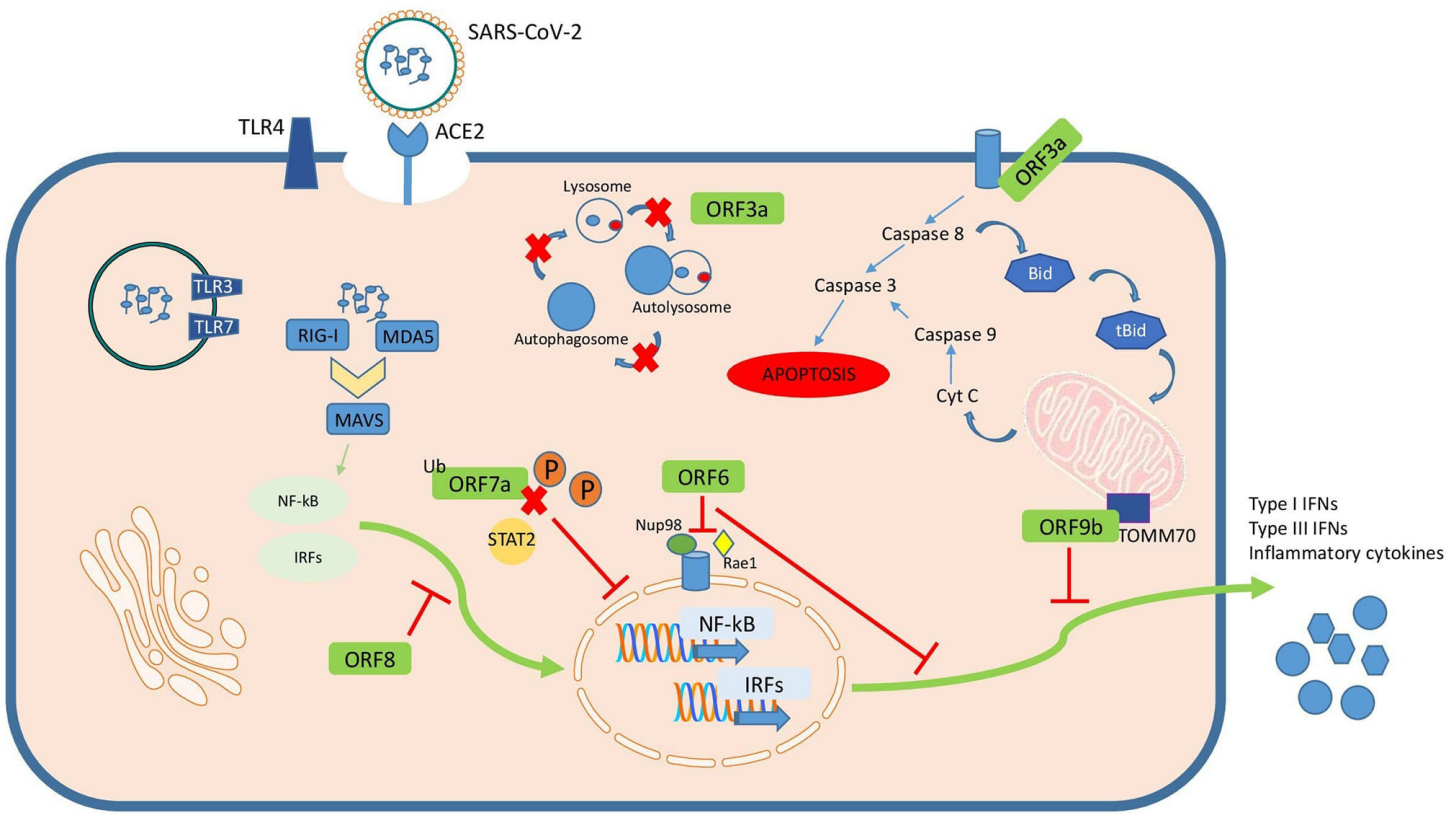
Summary of SARS-CoV-2 accessory proteins known mechanisms of action in the infected cell.

Accessory proteins are important virulence factors involved in different pathogenesis mechanisms during SARS-CoV-2 infection (**Figure 2**). Although some of them are not essential for replication their role remains obscure in terms of their influence on pathogenesis. Most of the roles ascribed to these accessory proteins are related to immune evasion mechanisms like inhibition of cytokine secretion by ORF9c, or counteracting type I IFN action by ORF3b, ORF6, ORF7a, ORF8 or ORF9b. Additionally, there are other important cellular mechanisms altered by these accessory proteins such as autophagy or apoptosis by ORF3a, mitochondrial function by ORF3d or inflammasome activation by ORF9b. Regulation of each sgRNA also determine expression and therefore interaction with cellular components. However, there are still many unknowns awaiting to be explored in order to better understand not only the current SARS-CoV-2 pandemic but also futures public health emergencies that might be caused by related coronavirus. Counteracting the action of those accessory proteins have been proposed as tentative targets for new drug development or repurposing.

## Author Contributions

NR and SZ-L wrote the first version of the manuscript. MM and JG reviewed, extended and edited the manuscript and figures. **Figure 1** was done by NR and reviewed by JG and **Figure 2** was done by NR. All authors contributed to the article and approved the submitted version.

## Funding

NR was a fellow funded by project grant 202020E170 from PTI Salud Global (CSIC). SZ-L was a Juan de la Cierva Incorporación fellow IJCI-2017-31382. This work was supported by grants 202020E170 from PTI Salud Global (CSIC) to MM and CV20-20089 of the Regional Government of Andalusia to JG.

## Conflict of Interest

The authors declare that the research was conducted in the absence of any commercial or financial relationships that could be construed as a potential conflict of interest.
